# A new secure image encryption algorithm based on a 5D hyperchaotic map

**DOI:** 10.1371/journal.pone.0242110

**Published:** 2020-11-12

**Authors:** Dejian Fang, Shuliang Sun

**Affiliations:** 1 College of Computer Science, Chongqing University, Chongqing, China; 2 School of Electronics and Information Engineering, Fuqing Branch of Fujian Normal University, Fuqing, China; Victoria University, AUSTRALIA

## Abstract

Image encryption is an effective method for protecting private images during communication. In this paper, a novel image encryption method is proposed based on a 5D hyperchaotic system. Since a 5D hyperchaotic system can generate more complex dynamic behavior than a low-dimensional system, it is used in this paper to generate pseudorandom number sequences. The generated sequences are processed to obtain new sequences. The randomness of the new sequences is improved by recombination and rearrangement. The experimental results and theoretical analysis show that the method possesses a large key space and can resist differential attacks, statistical analysis, entropy analysis, clipping attacks and noise attacks. Therefore, it is very secure and can be used for secure communication.

## 1 Introduction

With the rapid development of the Internet and communication technologies, researchers have focused increasingly more on information security. Images are known as one of the most important and popular multimedia technologies and are widely transmitted over the Internet. It is important to protect private images from hackers during communication. Image encryption is an effective method for protecting private images during communication, and many image encryption methods have been proposed [1–3]. Chaos is famous for its sensitivity to initial conditions and system parameters, pseudorandomness, ergodicity and reproduction. It is suitable for image encryption. Many chaotic image cryptosystems have been proposed [4–9]. Gao et al. [4] proposed a new nonlinear chaotic algorithm based on a power function and tangent function. The system parameters were obtained by experimental analysis. It is a one-time password system. Wang and Zhang [5] proposed a new color image encryption method based on bit permutations and correlated chaos. Heterogeneous a bit permutation process was adopted to reduce the computational cost and improve the permutation efficiency. An expanded XOR operation was also employed for the red (R), green (G) and blue (B) components of color images. Sun [6] proposed an image encryption scheme based on DNA operations and a chaotic map. A two-dimensional sine iterative chaotic map with an infinite collapse matrix was employed. An extended XOR operation was also applied to improve the security of the system. Liu and Wang [7] proposed a color image encryption scheme based on one-time keys. Image encryption using the DNA complementary rule and chaotic system was proposed in [8]. Wang et al. [9] proposed a chaotic image encryption algorithm based on a perception model. A fast image encryption algorithm based on the perceptron model was proposed in [10]. Wang and Gao proposed an image encryption algorithm based on matrix semi-tensor product theory and a Boolean network [11–12]. However, many image encryption methods employ low-dimensional chaotic systems [13–16]. Low-dimensional chaotic systems have a small key space and parameters. They are not safe enough to use as an image cryptosystem.

A hyperchaotic system is a better image cryptosystem than a low-dimensional chaotic system. A hyperchaotic system has more than one positive Lyapunov exponent. It generates more complex dynamic behavior and higher randomness than low-dimensional chaotic systems [17–21]. Ye and Wong [18] designed an image encryption scheme based on a time delay and a hyperchaotic system. A permutation function and double diffusion operations were executed in both the forward and reverse directions. Sun [19] proposed a novel hyperchaotic image encryption algorithm based on pixel-level scrambling, bit-level scrambling and DNA encoding. A 5-D hyperchaotic system was executed to generate chaotic sequences. Chen [20] proposed a fast chaos-based image encryption scheme with a dynamic state variable selection mechanism. Liu and Kadir [22] proposed color image encryption using bit-level permutations and a high-dimensional chaotic system. Sun et al. [23, 24] proposed a novel hyperchaotic image encryption method. Since a 5-D hyperchaotic system [25] has three positive Lyapunov exponents and generates more complex dynamic behavior than a low-dimensional system, we also adopt a 5-D hyperchaotic system to generate chaotic sequences in this paper. To eliminate the correlations between adjacent elements in chaotic sequences, the generated sequences are pretreated before being used for scrambling and diffusion. Compared with other encryption algorithms, the proposed method has advantages in efficiency and security.

In this paper, we propose a new image encryption method based on a 5D hyperchaotic system. First, a 5D hyperchaotic system is used to generate chaotic sequences. Then, chaotic sequences are preprocessed to obtain new sequences, which are used in the image confusion and diffusion processes.

The rest of this paper is organized as follows. The 5D hyperchaotic system and chaotic sequence generation are introduced in Section 2. The confusion and diffusion methods are described in Section 3. Section 4 discusses the experimental results and safety analysis. The conclusions are given in Section 5.

## 2 5D hyperchaotic system and chaotic sequence generation

### 2.1 5D hyperchaotic system

**Fig 1:**
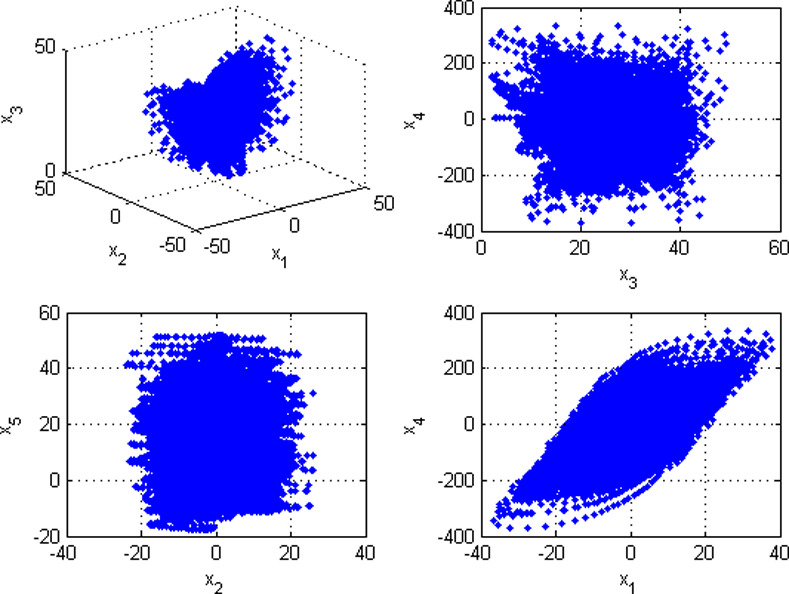
Sequence trajectories of system (1) with (a_1_, a_2_, a_3_, a_4_, a_5_, a_6_, a_7_) = (10, 1, 28, 8/3, 2, -1, 1).

A 5D hyperchaotic system [25] can be described as follows:

$$\left\{ \begin{array}{l} {\dot{x}}_{1}=a_{1}(x_{2}-x_{1})+x_{4}+a_{2}x_{5}\\ {\dot{x}}_{2}=a_{3}x_{1}-x_{2}-x_{1}x_{3}\\ {\dot{x}}_{3}=-a_{4}x_{3}+x_{1}x_{2}\\ {\dot{x}}_{4}=a_{5}x_{4}-x_{1}x_{3}\\ {\dot{x}}_{5}=a_{6}x_{1}+a_{7}x_{2} \end{array}\right.$$ (1)

where *a*_1_, *a*_2_, *a*_3_, *a*_4_, *a*_5_, *a*_6_ and *a*_7_ are system parameters. When *a*_1_ = 10, *a*_2_ = 1, *a*_3_ = 28, *a*_4_ = 8/3, *a*_5_ = 2, *a*_6_ = -1 and *a*_7_ = 1, the 5D hyperchaotic system is in a chaotic state and can produce five chaotic sequences. The sequence trajectories of system (1) are displayed in Fig 1.

### 2.2 Preprocessing of chaotic sequences

1. Calculate the initial values of system (1) as follows:

$$\left\{ \begin{array}{l} x_{1}(1)=\mathrm{mod}\left(\sum_{j=1}^{5}x_{j}^{0}+\frac{\sum_{z=1}^{MN}P(z)}{MN},1\right)\\ x_{i}(1)=\mathrm{mod}\left(x_{i-1}(1)+x_{i}^{0},1\right)\;i=2,3,4,5, \end{array}\right.$$ (2)

where $x_{1}^{0}$, $x_{2}^{0}$, $x_{3}^{0}$, $x_{4}^{0}$ and $x_{5}^{0}$ are the initial secret keys, and mod (*x*, *y*) represents the residue of *x* divided by *y*. The row and column of original plain image *P* are *M* and *N*.

2. System (1) is iterated *N*_0_ times to eliminate the transient response.

$$N_{0}=300+\mathrm{mod}\left(floor\left(\sum_{i=1}^{5}x_{i}^{0}\times 10^{10}\right),300\right)$$ (3)

where *floor* (*x*) returns the nearest integer value less than or equal to *x*.

3. System (1) continues to iterate *MN*/4 times to generate five real-number sequences: *X* = [*x*_1_, *x*_2_, …, *x*_*MN*/4_], *Y* = [*y*_1_, *y*_2_, …, *y*_*MN*/4_], *Z* = [*z*_1_, *z*_2_, …, *z*_*MN*/4_], *U* = [*u*_1_, *u*_2_, …, *u*_*MN*/4_], and *V* = [*v*_1_, *v*_2_, …, *v*_*MN*/4_].

4. Four sequences are chosen from the five chaotic sequences, and they are combined to become a new sequence with the length *MN*. There are 120 kinds of arrangement modes according to an arrangement study. For example, *A*_1_ = {*Y*, *Z*, *V*, *X*}, *A*_2_ = {*X*, *V*, *Z*, *U*}, *A*_3_ = {*V*, *X*, *Z*, *Y*} and *A*_4_ = {*U*, *Y*, *Z*, *V*}.

5. Sequences *A*_1_, *A*_2_ and *A*_3_ are rearranged to form new sequences *A*_1_’, *A*_2_’ and *A*_3_’, respectively. The processes of these rearrangements are demonstrated in Eqs 4 and 5.

$$\left[g,h\right]=\text{sort}\;\left(A_{4}\right)$$ (4)

$$A_{j}{}^{’}\left(i\right)=A_{j}\left(h\left(i\right)\right),$$ (5)

where sort is a sorting function; *i* = 1, 2, …, *MN*; *j* = 1, 2, 3; *g* is the new sequence; and *h* is the index value of *g*.

## 3 Confusion and diffusion methods

### 3.1 Confusion method

1. The sequence of *A*_1_^’^ is modified first as Eq 6.

$$A_{1}^{'}(i)=\mathrm{mod}(floor(abs(A_{1}^{'}(i)\times 10^{10})),MN)$$ (6)

where *i* = 1, 2, …, *MN*, and *abs*(*x*) is the absolute value of *x*.

2. Suppose *i* and *i*^’^ are the positions of plain image *P*. The corresponding confusion image is denoted as *P*^’^, and it is calculated as follows:

$$i^{'}=i+\mathrm{mod}\left(A_{1}^{'}(i)+P^{'}(i-1),MN+1-i\right)$$ (7)

where *i* = 1, 2, …, *MN*, and *P*^’^(0) is designated as the initial secret key.

3. The scrambling method is executed as

$$P^{’}\left(i\right)=P\left(i’\right),\;P(i^{’})=P\left(i\right)$$ (8)

where *P*^’^(*i*) is the scrambling image positioned at *i*, *P*(*i*’) and *P*(*i*) are the plain images positioned at *i*’ and *i*, respectively, for *i* = 1, 2,…, *MN*.

### 3.2 Diffusion method

1. The sequences $A_{2}^{'}$ and $A_{3}^{'}$ are modified as Eqs 9 and 10.

$$A_{2}^{'}(i)=\mathrm{mod}\left(floor(abs(A_{2}^{'}(i))\times 10^{15}),8\right)$$ (9)

$$A_{3}^{'}(i)=\mathrm{mod}\left(floor(abs(A_{3}^{'}(i))\times 10^{15}),256\right)$$ (10)

where $A_{2}^{'}(i)$ϵ[0, 7], $A_{3}^{'}(i)$ϵ[0, 255] and *i* = 1, 2, …, *MN*.

2. Convert decimal sequences P’ and $A_{2}^{'}$ into the corresponding binary sequences.

3. Sequence Q is obtained by Eq 11.

$$Q\left(r\right)=\text{CIRSFT}[P’\left(r\right),LSB(A_{2}^{'}\left(r\right)),A_{2}^{'}\left(r\right)]$$ (11)

where CIRSFT [*r*, *s*, *t*] represents the *t*-bit cyclic shift on binary sequence *r*. *LSB*(*t*) represents the smallest bit of *t*. The left cyclic shift or right cyclic shift will be decided by *s* = 0 or *s* = 1.

4. Convert the binary sequence *Q* into its decimal sequence.

5. Diffusion sequence *C* is obtained by Eqs 12–14.

$$sum=floor\left(\left(\sum_{j=1}^{5}x_{j}^{0}+\frac{P^{'}(0)}{256}\right)\times 10^{15}\right)$$ (12)

$$C(1)=A_{3}^{'}(1)⊕\mathrm{mod}\left(A_{3}^{'}(1)+Q(1),256\right)⊕\mathrm{mod}\left(sum,256\right)$$ (13)

$$C(i)=A_{3}^{'}(i)⊕\mathrm{mod}(A_{3}^{'}(i)+Q(i),256)⊕C(i-1)$$ (14)

where *Q*(*i*), *$A_{3}^{'}$*(*i*), *C*(*i*) and *C*(*i*-1) represent the decimal sequence value, chaotic sequence value, diffusion sequence value and previous diffusion value, respectively, and *i* = 2, 3, …, *MN*.

6. Convert *C* to a gray image *P*^”^. Finally, encrypted image *P*^”^ is obtained.

**Fig 2:**
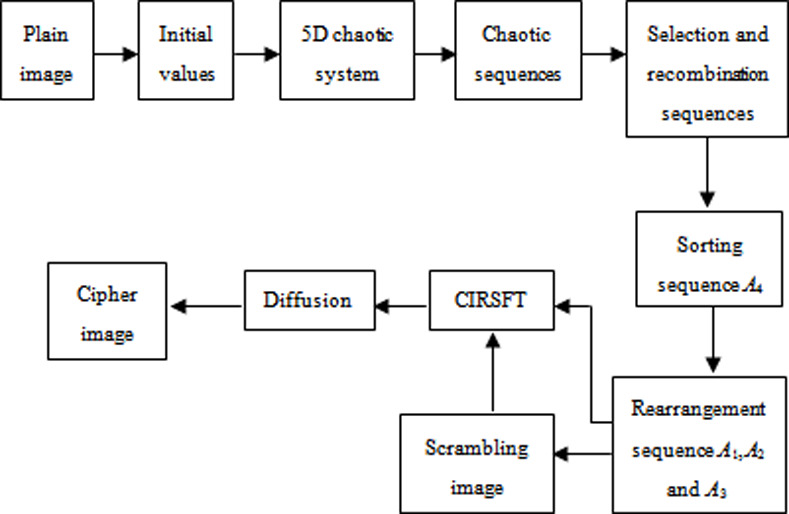
Flowchart of the image encryption procedure.

The flowchart of the image encryption procedure is displayed in Fig 2.

The decryption algorithm is the reverse process of the encryption algorithm.

## 4 Experimental results and safety analysis

**Fig 3:**
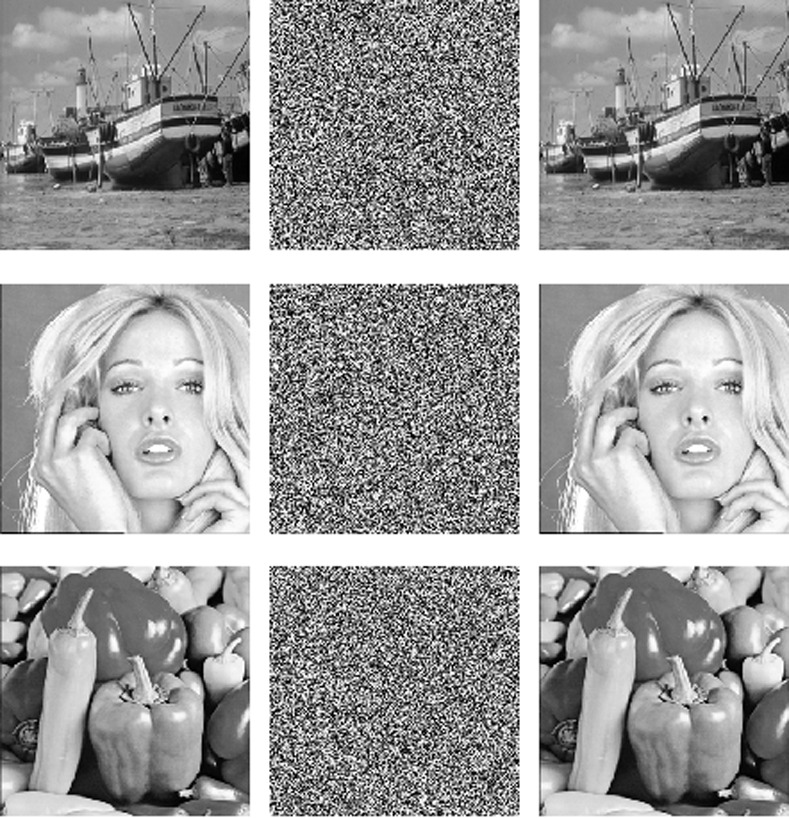
Encryption and decryption results. (a) Boat (b) Cipher image, (c) Decoded image, (d) Tiffany, (e) Cipher image, (f) Decoded image, (g) Pepper (h) Cipher image, (i) Decoded image.

In this paper, MATLAB 2010b is applied to execute the algorithm. The experiments are executed on a computer with a Windows 7 operating system, inter(R) Core (TM) i3-3220, 3.3 GHz and 8.00 GB RAM. The initial values of the 5D chaotic system are $x_{1}^{0}$ = 2.2356, *$x_{2}^{0}$* = 1.9057, *$x_{3}^{0}$* = 0.7468, *$x_{4}^{0}$* = 2.1577, *$x_{5}^{0}$* = 0.9723 and P^’^(0) = 128. The 256×256 gray images “Boat”, “Tiffany” and “Peppers” are used as the plain images. The plain, cipher and deciphered images are shown in Fig 3.

### 4.1 Key space analysis

In this paper, the key space is determined by the initial values of the 5-D hyperchaotic system {*x*_*i*_^0^_,_ *i* = 1, 2, …, 5}. If the precision of the system is 10^−15^, the key space is approximately (10^15^)^5^ = 10^75^≈2^249^. It is larger than 2^100^, so the proposed method could effectively resist a brute-force attack.

Other encryption schemes are compared with the proposed method in Table 1. It can be seen that the key space of the proposed method is much larger than those of Refs [26, 31] but smaller than that of Ref [19]. Although the key space in Ref [19] is larger than that of our scheme, it has more secret keys and is more complex.

**Table 1:** Key space results comparison with other methods.

Schemes	Ref [19]	Ref [26]	Ref [31]	proposed
Key space	10^90^	10^70^	10^58^	10^75^

### 4.2 Key sensitivity analysis

**Fig 4:**
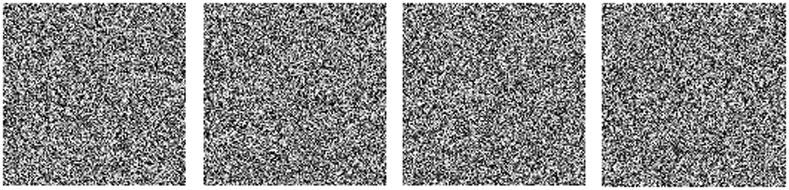
The key sensitivity test: (a) cipher image with x_1_ changed to x_1_+10^−15^; (b) cipher image with x_2_ changed to x_2_+10^−15^; (c) deciphered image with x_4_ changed to x_4_+10^−15^; (d) deciphered image with x_5_ changed to x_5_+10^−15^.

An excellent cryptosystem should be sensitive to the initial keys. Two completely different cipher images can be produced if a minor change (10^−15^) is made and the other four keys are unchanged. The cipher image also cannot be decrypted correctly if even a slight change (10^−15^) is made between the encryption and decryption keys. The key sensitivity test is shown in Fig 4.

Key *x*_1_ is changed to *x*_1_+10^−15^, and the cipher image is displayed in Fig 4(A). The value of *x*_2_ is modified to *x*_2_+10^−15^, and the corresponding cipher image is shown in Fig 4(B). The value of *x*_4_ is altered to *x*_4_+10^−15^ to decipher Fig 3(H), and the deciphered image is shown in Fig 4(C). Fig 4(D) is the deciphered image when *x*_5_ is altered to *x*_5_+10^−15^. Table 2 shows the differences between the different cipher and decipher images.

**Table 2:** Differences between the cipher and decipher images with minor key modifications.

Figures	Secret keys					Comparison with Fig 3(H)
	*x*_1_	*x*_2_	*x*_3_	*x*_4_	*x*_5_	
Fig 3(H)	$x_{1}^{0}$	$x_{2}^{0}$	$x_{3}^{0}$	$x_{4}^{0}$	_$x_{5}^{0}$_	-
Fig 4(A)	$x_{1}^{0}$+10^−15^	$x_{2}^{0}$	$x_{3}^{0}$	$x_{4}^{0}$	$x_{5}^{0}$	0.9965
Fig 4(B)	$x_{1}^{0}$	$x_{2}^{0}$+10^−15^	$x_{3}^{0}$	$x_{4}^{0}$	$x_{5}^{0}$	0.9974
Fig 4(C)	$x_{1}^{0}$	$x_{2}^{0}$	$x_{3}^{0}$	$x_{4}^{0}$+10^−15^	$x_{5}^{0}$	0.9963
Fig 4(D)	$x_{1}^{0}$	$x_{2}^{0}$	$x_{3}^{0}$	$x_{4}^{0}$	$x_{5}^{0}$+10^−15^	0.9954

It can be concluded that a small difference in the secret key will generate a completely different cipher image. It also cannot extract the correct deciphered image. If the secret key and plain image have a slight alteration, it is impossible to decrypt the plain image. The pixels differ by approximately 99.6% between the original image and the decrypted image.

### 4.3 Histogram analysis

**Fig 5:**
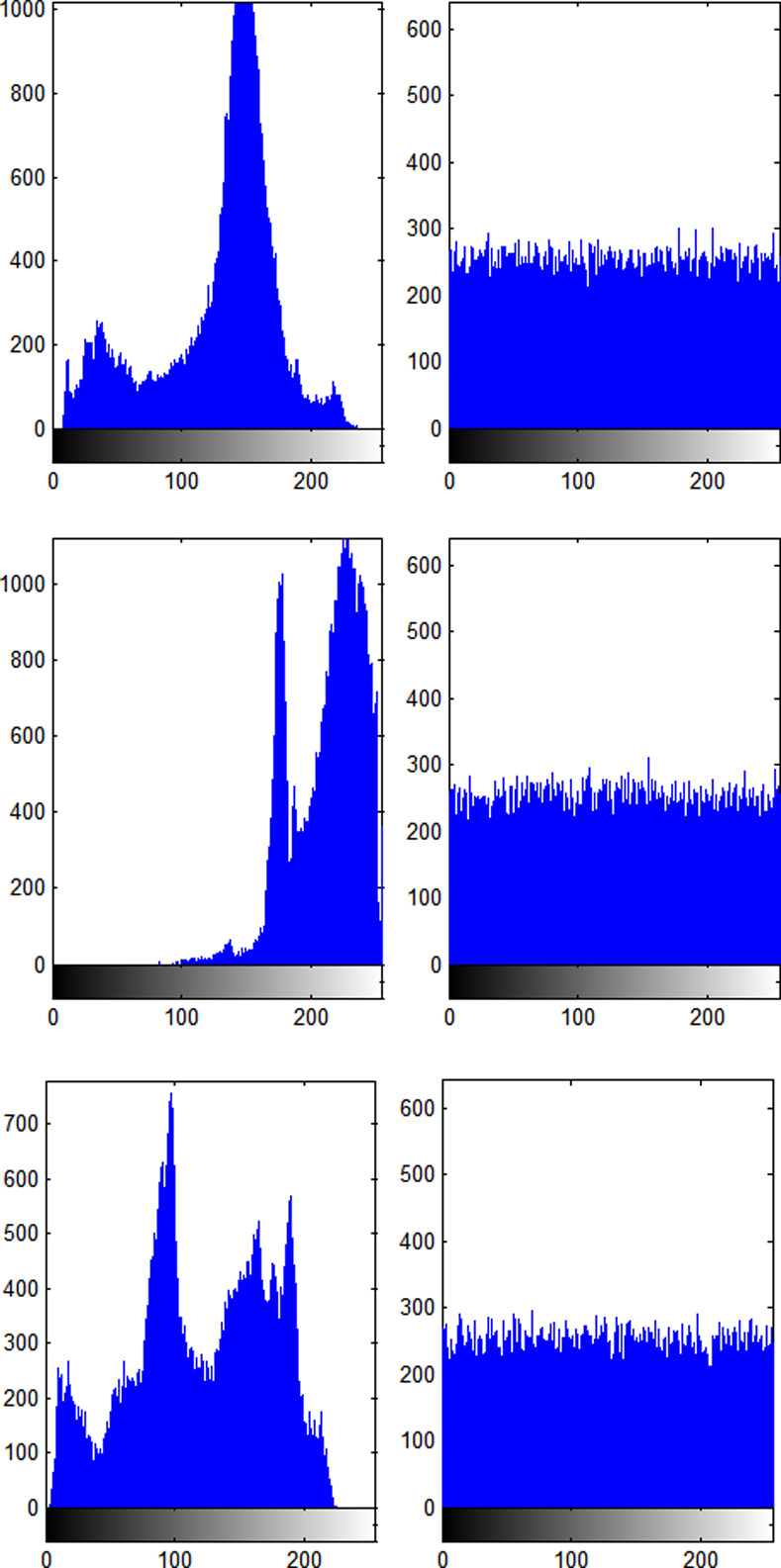
Histogram of the plain and cipher images. (a) Boat's histogram (b) Boat's cipher histogram, (c) Tiffany's histogram, (d) Tiffany's cipher histogram, (e) Pepper's histogram, (f) Pepper's cipher histogram.

The histogram of the cipher image should be as uniform as possible. In the proposed method, the histograms of the plain and cipher images of Boat, Tiffany and Pepper are displayed in Fig 5. It is shown that the plain image pixel values are centralized around some values; however, the corresponding cipher image pixel values are very smooth and even. Therefore, it makes a statistical attack ineffective.

The chi-square test [27, 28] is often used to measure the uniformity of the histogram. The chi-square results are listed in Table 3 for different cipher images.

**Table 3:** Chi-square test of the histograms.

Image	Boat	Tiffany	Pepper
$\chi_{test}^{2}$(255)	293.25 293.25 293.25		
$\chi_{test}^{2}$	245.37	265.62	256.29
Decision	Pass	Pass	Pass

It can be shown from Table 3 that all values generated by the proposed method are smaller than the theoretical value of 293.25 [29, 30]. It can be proven that the distribution of the histogram is flat, and the proposed method could pass the chi-square test.

### 4.4 Correlation analysis

The plain image pixel has a great correlation with its neighboring pixels. An excellent cryptosystem should reduce this correlation to close to zero. The correlation coefficient, *r*_*xy*_, between two adjacent pixels, *x* and *y*, is defined as:

$$E(x)=\frac{1}{N}\sum_{i=1}^{N}x_{i},\;D(x)=\frac{1}{N}\sum_{i=1}^{N}{\left(x_{i}-E(x)\right)}^{2}$$ (15)

$$\mathrm{cov}(x,y)=\frac{1}{N}\sum_{i=1}^{N}\left(x_{i}-E(x)\right)(y_{i}-E(y))$$ (16)

$$r_{xy}=\frac{\mathrm{cov}(x,y)}{\sqrt{D(x)D(y)}}$$ (17)

**Fig 6:**
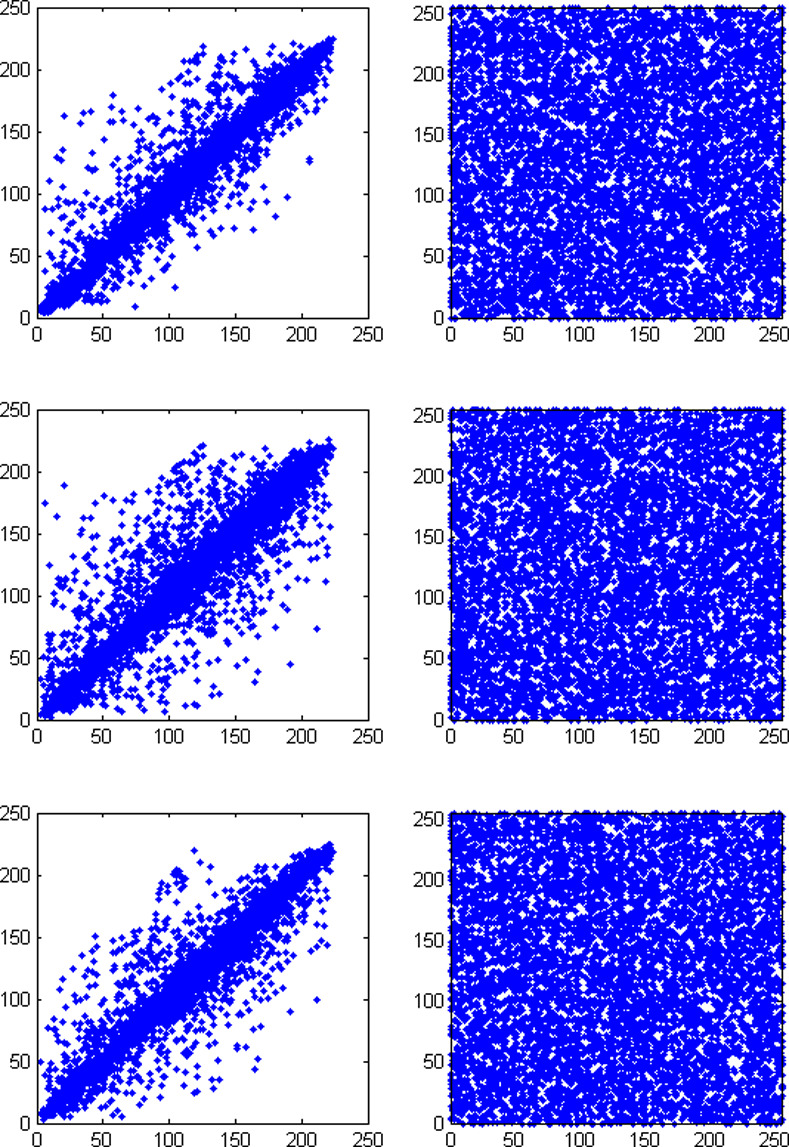
Correlation between the plain image Pepper and the cipher image in three directions. (a) Horizontal direction of the plain image, (b) Horizontal direction of the cipher image, (c) Vertical direction of the plain image, (d) Vertical direction of the cipher image, (e) Diagonal direction of the plain image, (f) Diagonal direction of the cipher image.

A total of 7225 pairs of adjacent pixels in the “Pepper” plain and cipher images are selected in the horizontal, vertical and diagonal directions. Fig 6 displays the correlation between two adjacent pixels in the plain image Pepper and the corresponding cipher image. It can be concluded that the pixels are highly correlated in the original image, while the correlation is considerably reduced in the cipher image.

Table 4 displays the correlation coefficients of the plain image Pepper and the cipher image.

**Table 4:** Correlation coefficients of the adjacent pixels in the Pepper image.

	Horizontal	Vertical	Diagonal
Plain image	0.9391	0.9700	0.9146
Cipher image	0.0068	-0.0054	0.0010

### 4.5 Information entropy analysis

Information entropy is the most important characteristic of randomness. If *m* is the information source, the information entropy can be defined as follows:

$$H(m)=-\sum_{i=1}^{L}p(m_{i})\log_{2}\;p(m_{i})$$ (18)

where *p*(*m*_*i*_) represents the frequency of symbol *m*_*i*_, and *L* denotes the number of *m*_*i*_. The information entropy data of the cipher image are shown in Table 5.

**Table 5:** Information entropy of the cipher image.

Image	Boat	Tiffany	Pepper
Proposed	7.9968	7.9972	7.9978
Ref. [19]	7.9967	7.9970	7.9976
Ref. [26]	7.9963	7.9966	7.9971
Ref. [31]	7.9965	7.9969	7.9973
Ref. [15]	7.9967	7.9970	7.9972

As displayed in Table 5, the information entropies of the cipher images are close to 8 bits. This also means that the ciphered image with our algorithm is very uniform. This result demonstrates that our method can resist entropy attacks. It can also be found that our algorithm is better than other similar methods.

### 4.6 Differential attack analysis

NPCR and UACI are two important parameters that are often employed to measure the sensitivity to plaintext [26]. These are defined as follows:

$$NPCR=\frac{1}{M\times N}\sum_{i=1}^{M}\sum_{j=1}^{N}D(i,j)\times 100\%$$ (19)

$$UACI=\frac{1}{M\times N}\sum_{i=1}^{M}\sum_{j=1}^{N}\frac{\left|C_{1}(i,j)-C_{2}(i,j)\right|}{255}\times 100\%$$ (20)

$$D(i,j)=\left\{ \begin{array}{l} 0,\;if\;C_{1}(i,j)=C_{2}(i,j)\\ 1,\;else \end{array}\right.$$ (21)

where *M* and *N* denote the width and height of the image, respectively, and *C*_1_ and *C*_2_ represent the ciphered images before and after one pixel of the plain image is modified, respectively.

The values of NPCR and UACI are shown in Table 6. It can be concluded that the proposed algorithm can effectively resist differential attacks.

**Table 6:** NPCR and UACI values for cipher images.

Image	NPCR (%)	UACI (%)
proposed	99.62	33.47
Ref. [26]	99.60	33.48
Ref. [19]	99.61	33.46
Ref. [31]	99.60	33.45
Ref. [15]	99.59	33.42

### 4.7 Clipping and noise attack analysis

**Fig 7:**
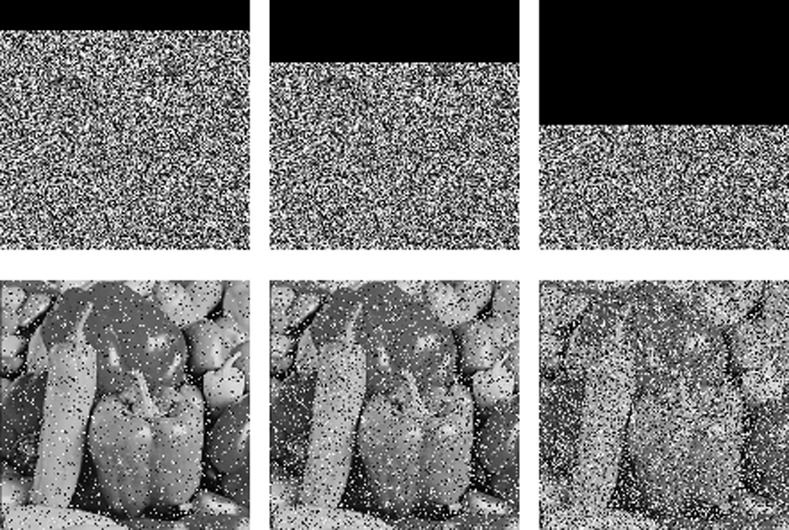
Recovery after different degrees of cropping attacks. (a) 1/8 cropped, (b) 1/4 cropped, (c) 1/2 cropped, (d) deciphered of (a), (e) deciphered of (b), (f) deciphered of (c).

A good cryptosystem should be designed to resist noise attacks and clipping attacks. The ciphered Pepper image of Fig 2(H) is cropped by 1/8, 1/4 and 1/2, and the decryption results are shown in Fig 7.

**Fig 8:**
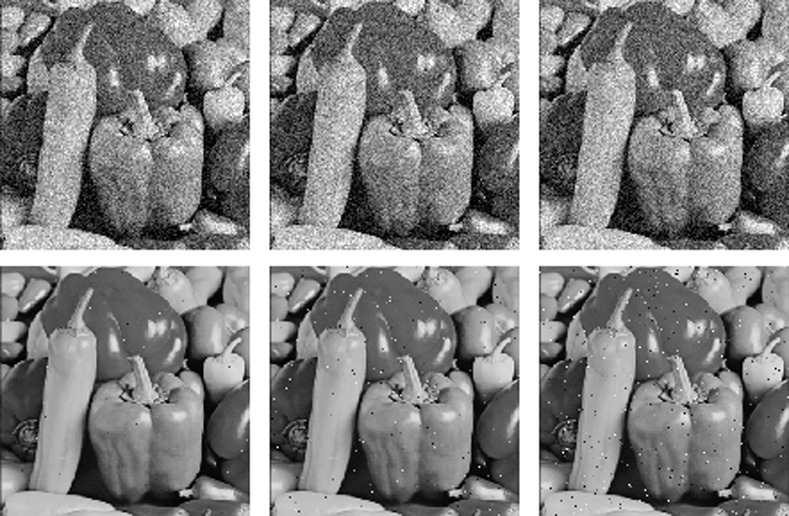
Decryption results with different noise. (a) Gaussian v = 0.001, (b) Gaussian v = 0.005, (c) Gaussian v = 0.01, (d) Salt and pepper d = 0.001, (e) Salt and pepper d = 0.005, (f) Salt and pepper d = 0.01.

Salt and pepper noise and white Gaussian noise are added to the ciphered Pepper image in Fig 2(H), and the deciphered images are shown in Fig 8. When the variance of the white Gaussian noise is increased from 0.001 to 0.01, more noise points appear in the deciphered image, but the deciphered image is still recognizable. Similar results were obtained for the salt and pepper noise.

The results prove that the proposed method effectively resists cropping and noise attacks.

The peak signal-to-noise ratio (*PSNR*) is used to measure the ability of the method to resist noise and data loss [32]. It is adopted to measure the difference between plain image *I* and cipher image *I*’. The PSNR is defined as follows:

$$PSNR=20\log_{10}\frac{255}{\sqrt{MSE}}$$ (22)

$$MSE=\frac{1}{MN}{\sum_{i=1}^{M}\sum_{j=1}^{N}\left(I_{ij}-I_{ij}^{'}\right)}^{2}$$ (23)

The higher the value of the PSNR is, and the smaller the difference between I and *I*’. The results are shown in Tables 7 and 8.

**Table 7:** PSNR values of different schemes with different percentages of salt and pepper noise.

Method	Noise density (%)					
	0.01	0.05	0.1	0.5	1	5
Ref. [32]	41.40	34.17	30.93	24.03	21.18	14.32
Ref. [33]	9.52	8.93	8.62	8.56	8.55	8.55
Ref. [34]	42.69	36.84	31.53	28.71	9.29	18.84
Proposed	59.69	56.79	52.46	48.51	43.59	38.16

**Table 8:** PSNR values of different schemes with different percentages of data loss.

Method	Data loss (%)				
	1/32	1/16	1/8	1/4	1/2
Ref. [32]	20.76	18.51	15.93	11.15	8.72
Ref. [33]	8.615	8.568	8.554	8.550	8.548
Ref. [34]	24.37	20.63	17.64	14.61	11.61
Proposed	41.64	39.28	35.84	33.12	30.07

It can be seen from Tables 7 and 8 that the proposed algorithm obtains higher PSNR values than those in [32–34] when decrypting images under noise and cropping attacks. Therefore, the proposed scheme is superior to the comparative ones.

### 4.8 Classical types of attacks

There are four classical types of attacks: ciphertext only, known plaintext, chosen ciphertext, and chosen plaintext. If a cryptosystem can resist a chosen plaintext attack, then it will be able to resist other attacks [3].

The proposed method is sensitive to initial values *x*_*i*_^0^ (i = 1, 2, 3, 4, 5) and the plain image. If one of them is changed, then the generated chaotic sequences will be completely different. The ciphered value not only connects to the confused pixel but also connects to the former confused pixel value and former ciphered value. This means that different ciphered values have different former confused values and different ciphered values. Therefore, the proposed scheme could defend against chosen plaintext attacks.

### 4.9 Encryption time analysis

The results of the comparison are shown in Table 9. As shown in Table 9, the proposed method requires the least encryption time compared with the other algorithms. Thus, our proposed method has better performance than other schemes.

**Table 9:** Comparison of encryption times (seconds).

Image size	Ref [19]	Ref [26]	Ref [31]	Proposed
128×128	1.93	1.68	1.90	1.28
256×256	7.72	6.72	7.59	5.14
512×512	31.58	26.88	30.35	20.56

## 5 Conclusion

In this paper, a novel image encryption scheme is proposed based on a 5D hyperchaotic system. First, chaotic sequences are produced by a 5D hyperchaotic system based on initial secret keys. Then, the chaotic sequences are preprocessed to obtain new chaotic sequences. They are modified so that they can be used in confusing and diffusing the image. A cycle shift is executed to improve the security of the cryptosystem. The experimental results and theoretical analysis demonstrate that the method has a large key space and resists differential attacks, brute-force attacks, statistical attacks, clipping attacks and noise attacks. Therefore, it is a high-security method that can be used in practical applications.

## Supporting information

S1 Material (RAR)
